# No link between the prevalence of hepatitis E virus infection and the diagnosis of schizophrenia

**DOI:** 10.1007/s15010-022-01871-2

**Published:** 2022-06-16

**Authors:** Claudia Beisel, Sven Pischke, Maria Mader, Steffen Moritz, Daniel Schöttle, Daniel Lüdecke

**Affiliations:** 1 I. Department of Medicine, University Medical Center Hamburg-Eppendorf, Hamburg, Germany; 2 German Center for Infection Research (DZIF), University Medical Center Hamburg-Eppendorf, Lübeck-Borstel-Riems, Hamburg, Germany; 3 Department of Psychiatry and Psychotherapy, University Medical Center Hamburg-Eppendorf, Hamburg, Germany; 4 Department of Psychiatry, Psychotherapy and Psychosomatics, Asklepios Clinic Hamburg-Harburg, Hamburg, Germany

Infection with the Hepatitis E Virus (HEV) may cause strong inflammation of the liver and represents one of the most common causes of acute hepatitis in the world. Extrahepatic manifestations, such as neurological, hematological, or renal manifestation as well as several autoimmune phenomena have been reported in association with HEV infection [1]. There are only limited data about the association between HEV infection and psychiatric disorders. Few epidemiological studies from Taiwan, China, and Germany, demonstrated high anti-HEV IgG seroprevalence rates in psychiatric patients [2–5]. However, these studies often lack adequate control cohorts, making it difficult to adequately classify seroprevalence rates. Systematic studies that address the risk of psychiatric patients acquiring HEV infection, or if a previous HEV infection may trigger psychiatric diseases are missing.

Here, we present a cohort of 39 patients with the initial diagnosis of acute schizophrenia. Serum samples and cerebrospinal fluid (CSF) were tested for HEV by PCR (*Altona diagnostics Realstar* HEV RT-PCR kit, Altona diagnostics, Hamburg, Germany) and anti-HEV IgG (Wantai assay, Wantai, Bejing, China) to define the prevalence of acute or previous HEV infection in this psychiatric patient cohort. CSF was available in 32 patients (82%). Patients were identified between January 2016 and March 2018 at the Department of Psychiatry of the University Medical Center Hamburg-Eppendorf and included in this monocenter study. The diagnosis of acute schizophrenia was established by a highly specialist psychiatrist. Patients’ age ranged from 18 to 52 years, with a mean age of 31 years. With 21 of 39 patients (54%), there was a slightly higher proportion of female individuals.

Since 35 patients (90%) were tested negative for HEV serum Immunoglobulin G (IgG), in four patients HEV IgG was found to be positive (10%), indicating a recent or prior HEV infection. The seroprevalence of anti-HEV IgG was higher in males compared to females (22% versus 0%, *p* = 0.01). To exclude recent, ongoing HEV infection, we performed HEV polymerase chain reaction (PCR) in serum and CSF samples. HEV ribonucleic acid (RNA) was not detected in any of the serum or CSF samples, which allowed us to exclude active HEV infection in all of the 39 patients (see Table 1).

**Table 1:** Patient characteristics

Characteristic	Total study population *n* = 39 (100%)	Females *n* = 21 (54%)	Males *n* = 18 (46%)
Age in years; mean, range	31; 18–52	29; 19–41	30; 18–52
HEV IgG, serum			
Available, *n* (%)	39 (100)	21 (100)	18 (100)
Negative, *n* (%)	35 (90)	21 (100)	14 (78)
Positive, *n* (%)	4 (10)	0 (0)	4 (22)
HEV RNA, serum			
Available, *n* (%)	39 (100)	21 (100)	18 (100)
Negative	39 (100)	21 (100)	18 (100)
Positive	0 (0)	0 (0)	0 (0)
HEV RNA, CSF			
Available, *n* (%)	29 (74)	17 (81)	12 (67)
Negative	29 (100)	17 (100)	12 (100)
Positive	0 (0)	0 (0)	0 (0)

A total number of 39 patients with the diagnosis of acute schizophrenia were included in this study. There were 21 female patients. The mean age of the study population was 31 years. In all patients serum samples were available. Four patients were tested positive for HEV IgG (10%). HEV RNA was not detected in any of the serum samples. Cerebrospinal fluid (CSF) was available in 29 patients (74%). In none of the serum or CSF samples, HEV RNA was detected by PCR

Taken together, the overall HEV IgG seroprevalence of the presented cohort was 10%. According to previously published cohorts, the HEV IgG seroprevalence in German healthy controls ranges from 15.3 to 18.6% in recent years [6]. Unlike previous studies [2–5], our database is a well-defined cohort of patients with acute schizophrenia at the time point of initial diagnosis. None of these patients tested positive for HEV RNA and the seroprevalence rate was in no way elevated in comparison to healthy individuals.

In conclusion, the presented data provided no evidence for an association between the diagnosis of HEV infection and an acute episode of schizophrenia and therefore we cannot confirm the previous studies, which unfortunately did not refer to comparative cohorts with healthy people.

## Data Availability

Data storage is performed by the *Universitätsklinikum Hamburg-Eppendorf*. Data are available upon request from the corresponding author and can be shared after confirming that data will be used within the scope of the originally provided informed consent.

## References

[1] Pischke S, Hartl J, Pas SD, Lohse AW, Jacobs BC, van der Eijk AA (2017). Hepatitis E virus: Infection beyond the liver?. J Hepatol.

[2] Cong W, Meng Q-F, Li B, Ma F-L, Qian A-D, Wang X-Y, Yu C-Z, Zhu X-Q (2014). Seroprevalence of hepatitis E virus infection in psychiatric patients and control subjects in Shandong Province, eastern China. Int J Infect Dis.

[3] Reinheimer C, Allwinn R, Berger A (2012). Hepatitis E: are psychiatric patients on special risk?. Med Microbiol Immunol.

[4] Cheng P-N, Wang R-H, Wu I-C, Wu J-C, Tseng K-C, Young K-C, Chang T-T (2007). Seroprevalence of hepatitis E virus infection among institutionalized psychiatric patients in Taiwan. J Clin Virol.

[5] Xue Y, Sun X, Li Y, Liu X, Dong C (2013). Increased risk of hepatitis E virus infection in schizophrenia. Adv Virol.

[6] Faber M, Willrich N, Schemmerer M, Rauh C, Kuhnert R, Stark K, Wenzel JJ (2018). Hepatitis E virus seroprevalence, seroincidence and seroreversion in the German adult population. J Viral Hepatitis.

